# Digital Health Interventions to Prevent Posttraumatic Arthritis After Traumatic Knee Injury: Scoping Review

**DOI:** 10.2196/99607

**Published:** 2026-10-28

**Authors:** Josh Cosgrove, Rodrigo Rabello, Emma Dunphy, William Marsh, Dylan Morrissey

**Affiliations:** 1 Sport and Exercise Medicine, Queen Mary University of London, Mile End Road, London, England, E1 4NS, United Kingdom, 1 02078825016; 2 Physiotherapy Department, Barts Health NHS Trust, London, England, United Kingdom; 3 Department of Primary Care & Population Health, University College London, London, England, United Kingdom; 4 Electronic Engineering and Computer Science, Queen Mary University of London, London, England, United Kingdom

**Keywords:** digital health intervention, traumatic knee injury, posttraumatic osteoarthritis, mobile health, mHealth, eHealth, OPTIKNEE

## Abstract

**Background:**

Traumatic knee injuries (TKI) are common and are associated with a 4 to 6 times increased risk of posttraumatic knee osteoarthritis (PTOAK) over the subsequent 15- to 20-year period. There is clear evidence that the risk can be reduced, as per the 2022 OPTIKNEE recommendations, but long-term care availability is limited. Digital health interventions (DHIs), such as wearable devices, telehealth innovations, and mobile apps, have the potential to deliver long-term musculoskeletal care. DHIs can be scaled up to support health care delivery by improving efficiency and accessibility.

**Objective:**

This study aimed to evaluate existing DHIs against the OPTIKNEE consensus guidelines for PTOAK prevention and to investigate adoption into practice.

**Methods:**

A search of 7 online academic databases and the gray literature was completed from inception to June 3, 2025, complemented by hand searching government, charity, and university websites for reports and technical prototype papers concerning DHIs that can support care after TKIs. The content from each DHI was mapped against the 7 OPTIKNEE recommendations as follows: exercise, goal setting, patient-reported outcome measures, functional assessment, strength assessment, information provision, and overall clinical outcome. DHIs were then evaluated against the health-technology pathway to identify their stage of development. Finally, adoption into routine clinical practice was analyzed using Normalization Process Theory (NPT), which considers 4 domains: coherence, cognitive participation, collective action, and reflexive monitoring.

**Results:**

There were 81 reports, 53 peer-reviewed and 28 from other sources, concerning 49 distinct DHIs. They were designed for injuries of the anterior cruciate ligament (ACL, n=12); ACL±meniscus (n=15); meniscus (n=3); ACL or meniscus (n=2), bone (n=2), patella dislocation (n=1); and 14 were nonspecific. Across the 49 DHIs, 25 were classified as mobile health, 19 as telerehabilitation, 4 as eHealth, and 1 as both telerehabilitation and mobile health. No DHIs addressed all the OPTIKNEE recommendations; however, the 8 most complete reported 4 out of 7 components, including exercise, information provision, patient-reported outcome measures, goal setting, and overall patient outcomes. Only 1 DHI reported a remote functional assessment, and self-assessed strength evaluations were not reported in any DHI. NPT analysis typically demonstrated low DHI adoption across the 4 domains, and positive responses across the 4 NPT domains varied across the different health technology pathway stages. The DHI with the highest adoption into routine practice, according to NPT, was “getUbetter,” with 56% (n=9) positive scores.

**Conclusions:**

There are many available, or developing, DHIs, but none include the full content recommended by OPTIKNEE to reduce the risk of PTOAK. Furthermore, there is negligible evidence of DHIs being adopted into usual care irrespective of the stage of DHI development. There is a clear need to develop guideline-compliant DHIs to support effective prevention.

## Introduction

Individuals who experience a traumatic knee injury (TKI) often develop posttraumatic knee osteoarthritis (PTOAK) [1]. TKIs are common and occur in 720 to 1800 per 100,000 people annually [2] and tend to result in poor health outcomes, including a 6-fold increase in risk of osteoarthritis [3]. A cohort study found that out of 1145 knee injuries, 733 (64%) occurred in men aged 20 to 29 years [4]. At present, an estimated 5.4 million people live with knee osteoarthritis in the United Kingdom [5]. Those with PTOAK experience lifelong consequences such as pain, loss of function, obesity, fear, and reduced knee-related quality of life (QoL) [6]. The 2022 OPTIKNEE international consensus provides high-quality evidence on how to optimize knee health after a TKI [7]. The guidance details how to support individuals who have had a single or multistructure injuries, such as an anterior cruciate ligament (ACL) tear, meniscus tear, intraarticular tibiofemoral fracture, or patella dislocation with concomitant chondral lesions.

Recent technological advances have led to a boom in the use and acceptance of digital technologies as part of health care transformations aiming to increase capacity and access while controlling costs [8]. Despite strong claims, there is limited evidence that digital health interventions (DHIs) are adopted at scale or that they provide effective patient education and self-management interventions for people with musculoskeletal conditions, either in isolation or in combination with traditional care [9]. Digital tools have the potential to be scaled up to improve health care delivery by improving efficiency, accessibility, safety, and effectiveness [10]. This is recommended in the 10-year health plan for England, which aims to move much of health care from analog to digital [11]. A wide variety of DHIs are available, from smartphone apps to telemedicine platforms, which can support treatment and self-management [12]. A systematic review that evaluated DHIs found them to be effective in the medium term for reducing pain and improving physical function for individuals with knee and hip osteoarthritis [13]. DHIs have the power to increase access to health care; however, social determinants of health, such as digital literacy, are a major barrier to realizing the full benefits that DHIs have to offer [14,15]. Other barriers include low literacy [16], poor internet reliability [17], and being unable to afford the cost of phones or data [18]. DHIs can effectively support patients after a TKI, with a successful DHI likely needing to meet the OPTIKNEE recommendations to reduce PTOAK risk.

Despite the proliferation of DHIs [19], it is not clear whether these are being integrated, adopted, and used in routine practice. Normalization process theory (NPT) [20] is a useful framework for understanding how complex interventions become embedded into routine practice because it can be applied across different contexts [21] and provides a method of accurately explaining complex implementation processes [22]. It has been successfully deployed for DHIs such as telehealth consultations for asthma care [23] and clinical decision support systems [19,20].

The aim of this scoping review was to explore the quality, relevance, and adoption of DHIs available to support individuals after a TKI and reduce PTOAK risk. The objectives were to compare the DHIs against the OPTIKNEE consensus guidelines [7] and to investigate whether these DHIs have been adopted into routine practice using the NPT framework. The outcomes will guide clinical service delivery and inform future research on supporting patients after TKI.

## Methods

### Study Protocol

This review followed the Joanna Briggs Institute guidance for scoping reviews [24] and was reported according to the PRISMA-ScR (Preferred Reporting Items for Systematic Reviews and Meta-Analyses Extension for Scoping Reviews) checklist [25]. The protocol was published on the Open Science Framework (June 11, 2025) [26]. We did not fully address the presence of efficacy, proof of concept, regulatory approval, and link to evidence as initially planned. During the data analysis, it became clear that a focus on the OPTIKNEE mapping and NPT analysis was sufficient to give a clear and robust understanding of the literature regarding DHIs in this context.

### Data Sources and Search

Searches were conducted between May and June 2025 in both academic (PubMed, CINAHL, EMBASE, MEDLINE, IEEE Xplore, TRIP, and Web of Science) and nonacademic databases (NHS Digital Playbook, Innovate UK, iMedicalApps [27], Medicines and Healthcare Products Regulatory Agency [MHRA], Food and Drug Administration [FDA], Google Scholar, Queen Mary Research Online, Arthritis UK, and the University of British Columbia Open Collections). The search script was developed with the research team, based on key operational definitions (Table 1), including a health sciences librarian (Table 2; full search script in the Multimedia Appendix 1), and limiting it to manuscripts published from 2010 onward. Records were uploaded to Covidence (Veritas Health Innovation Ltd) [28] and duplicates removed. Dual title and abstract screening was conducted by the lead author and one other reviewer, with eligible full-texts being retrieved and screened similarly. The inclusion and exclusion criteria can be found in Textbox 1.

**Table 1.** Operational definitions.

Term	Definition
Digital health intervention	A health intervention delivered through digital tools or communication technologies, which collect, store, share, and analyze health information for the purpose of improving patient health and care. This could be through wearable devices, mobile apps, text with smartphones, or telehealth [29].
Traumatic knee injury	An isolated or combined traumatic tibiofemoral or patellofemoral (eg, ligament, meniscal, cartilage, subluxation, dislocation, and fracture) injury [1].
Posttraumatic knee osteoarthritis	Structural or symptomatic osteoarthritis that develops after sustaining a traumatic knee joint injury [7].

**Table 2.** Search terms.

Construct	Keywords
Injury	“knee dislocation” OR “acl” OR “anterior cruciate ligament” OR “meniscus” OR “knee fracture” OR “knee injury”
Digital health intervention	“wearable device” OR “mobile application” OR “mobile applications” OR “mobile apps” OR “mobile app” OR “internet” OR “mobile phone” OR “smart phone” OR “telemedicine” OR “technology” OR “mhealth” OR “telehealth” OR “emedicine” OR “mobile health” OR “cell phone” OR “digital health technology” OR “digital health”
Treatment and management	“outcome assessment” OR “monitoring” OR “kinesiotherapy” OR “signposting” OR “advice” OR “education” OR “telerehabilitation” OR “exercise” OR “rehabilitation” OR “patient reported outcome measure” OR “diet”

Textbox 1. **Inclusion and exclusion criteria**

**Inclusion criteria**

- Reports that include a digital health intervention (DHI), including but not limited to telemedicine, mobile health, telerehabilitation, mobile apps, telehealth, or wearable devices (including virtual reality).
- Reports that include patients who have undergone a traumatic knee injury (TKI), including but not limited to anterior cruciate ligament (ACL) rupture (preoperative or postoperative), meniscus tear, intraarticular tibiofemoral fracture, or patella dislocation.
- Reports including at least one of the following delivered via a DHI: outcome measures, weight management strategies, physical monitoring (including wearables), exercise prescription, signposting, or education.

**Exclusion criteria**

- Reports that do not include a TKI.
- Reports that do not include a DHI that could be used with people who have experienced a TKI.
- Reports including participants with osteoarthritis or participants who have undergone joint arthroplasty.
- Reports not reporting at least one of the following: outcome measures, weight management strategies, physical monitoring (including wearables), exercise prescription, signposting, or information provision/education.
- Reports involving the use of exoskeletons or electromyography.

### Data Extraction

For data charting, we used Elicit, the AI-assisted research tool [30], to identify initial data, which was then checked and confirmed by one author (JC) and supplemented where required. Each report was uploaded, and prompts were provided of the extraction of relevant data. Elicit data extraction has been shown to be of similar accuracy to human reviewers (81.4% vs 86.7% accuracy, respectively) [31]. Initial data extraction included study year, methods, DHI classification, and injury type. Then, from each DHI identified, we determined which of the key OPTIKNEE consensus domains it referenced [7]: exercise, education/advice, functional assessment, strength assessment, patient-reported outcome measures, patient goals, and outcomes. Elicit was used to provide an initial interpretation of the data extracted for each validated NPT question (Multimedia Appendix 1, Table 5), also being cross-checked by JC and any queries being discussed with the wider research team.

### Analysis Framework

The content from each DHI was mapped against the key OPTIKNEE [7] domains (Table 3). This process involved exploring the content of each DHI to verify if it reported information provision, exercises prescription, self-assessed functional assessment, self-assessed strength assessment, patient-reported outcome measure (PROM) delivered via the DHI, goal setting, or overall patient outcome. If the DHI reported any of the OPTIKNEE domains, then a “yes” score was given; if it was unclear from any of the reports, then an “unclear” score was given; and if there was no mention in the report, then a “no” score was given.

**Table 3.** The digital health interventions (DHIs) compared against the OPTIKNEE domains.

DHI	OPTIKNEE domains							Yes (%)
	Exercise	Information provision	Function	Strength	PROMS^a^	Goals	Outcome	
Orthopy^b^	☑^c^	☑	☒^d^	☒	☑	☒	☑	57
Rehabilitation cloud platform	☑	☑	☒	☒	☑	☒	☑	57
SKYRE	☑	☑	☒	☒	☑	☒	☑	57
KneeCare^e^	☑	☑	☒	☒	☑	⍰^f^	☑	57
No name (Levinger et al, 2017)	☑	☑	☒	☒	☑	☒	☑	57
No name (Martin et al, 2024)^b^	☑	☑	☒	☒	☑	☒	☑	57
getUbetter^e^	☑	☑	☒	☒	☑	☑	☒	57
TRAK-ACL	☑	☑	☒	☒	☑	☑	☒	57
Sword Health^e^	☑	☑	☒	☒	☒	☑	☒	42
Limber Health DETA^e^	☑	☒	☒	☒	☑	☒	☑	42
KC@H^b^	☑	⍰	⍰	☒	☑	☒	☑	42
No name (Brewer et al, 2022)^b^	☑	☑	☒	☒	☒	☑	☒	42
No name (Li, 2022)	☑	☒	☒	☒	☑	☒	☑	42
Doct’Up	☑	⍰	☒	☒	☒	☑	☒	28
BANG^g^	☒	☑	☒	☒	☒	☑	☒	28
QoC Health Inc	☒	☒	☒	☒	☑	☒	☑	28
SOAR^e^^,^^h^	☑	☑	☒	☒	☒	☒	☒	28
KNEESUP	☑	☒	☒	☒	☑	☒	☒	28
Orthelligent^b^	☑	☒	☑	☒	☒	☒	☒	28
Sana	☑	☒	☒	☒	☒	☑	☒	28
Mawendo	☑	☑	☒	☒	☒	☒	☒	28
ACL-Well^b^	☑	☑	☒	☒	⍰	☒	⍰	28
Khymeia System	☑	⍰	☒	⍰	☑	☒	☒	28
CBPT-ACLR^i^	☒	☑	☒	☒	⍰	☑	⍰	28
ACL^j^ Rehab	☑	☑	☒	☒	☒	☒	☒	28
HJT Software^b^	☑	☑	☒	☒	☒	☒	☒	28
CHES^e^	☒	☒	☒	☒	☑	☒	☑	28
Phio^e^	☑	⍰	☒	☒	☒	☑	☒	28
No name (Nguyen et al, 2017)	☒	☒	☒	☒	☑	☒	☑	28
No name (Tipprom et al, 2018)^e^	☑	☒	☒	☒	☑	☒	☒	28
No name (Kordatos and Stavrakis, 2018)	☑	☒	☒	⍰	☒	☑	☒	28
Nefitness^e^	☑	☒	☒	☒	⍰	☒	⍰	14
NeoRehab	☑	⍰	☒	☒	☒	☒	☒	14
Physitrack^e^	☑	☒	☒	☒	☒	☒	☒	14
Upscaler^b^	☑	☒	☒	☒	☒	☒	☒	14
FitBit^e^	☒	☒	☒	☒	☒	☑	☒	14
exoRehab	☑	☒	☒	☒	☒	☒	☒	14
Joymotion	☑	☒	☒	☒	☒	☒	☒	14
UIN-CARE Home 1	☑	☒	☒	☒	☒	☒	☒	14
Microsoft Kinect^e^	☑	☒	☒	☒	☒	☒	☒	14
No name (Litchfield and Buttress, 2019)	☒	☑	☒	☒	⍰	☒	☒	14
No name (Hadley et al, 2019)	☑	☒	☒	☒	⍰	☒	☒	14
No name (Wang, 2025)	☑	☒	☒	☒	☒	☒	☒	14
No name (Zadrapova et al, 2022)	☑	☒	☒	☒	☒	☒	☒	14
No name (Tsvyakh et al, 2021)^e^	☑	☒	☒	☒	☒	☒	☒	14
No name (Wang et al, 2023)^b^	☑	☒	☒	☒	☒	☒	☒	14
No name (Ling and Yan, 2022)	☑	☒	☒	☒	☒	☒	☒	14
No name (Kalofonos et al, 2024)^b^	☑	☒	☒	☒	☒	☒	☒	14
No name (Anthony et al, 2020)	☒	☒	☒	☒	☒	☑	☒	14
Yes (%)	84	37	2	0	35	24	24	

a PROM: patient-reported outcome measure.

b The DHI was released after the OPTIKNEE recommendations were published.

c The OPTIKNEE domain was provided in the content of the DHI.

d The OPTIKNEE domain was not present in the content of the DHI.

e A DHI that is not specifically designed for people who have had a TKI, but it has the potential to be used with a person who has had a TKI.

f It is not clear if the OPTIKNEE domain was provided in the content of the DHI.

g BANG: back in the game.

h SOAR: SOAR: stop osteoarthritis.

i CBPT-ACLR: Cognitive behavioral–based physical therapy anterior cruciate ligament reconstruction.

j ACL: anterior cruciate ligament.

NPT provides a framework for data analysis [32] and previous evidence supports the use of NPT for evaluating and understanding implementation processes [33]. The NPT framework contains 16 questions split into 4 domains: coherence, cognitive participation, collective action, and reflexive monitoring [20] (Multimedia Appendix 1, Table S5). For each question in the NPT framework, we gave an answer of “yes,” “no,” “maybe,” or “not reported,” and this was then color coded in the analysis. Following this, relevant quotes were extracted from the reports to evidence the decisions made (Tables 4-6).

**Table 4.** Normalization process theory (NPT) results for digital health intervention (DHIs) at the market stage of the health technology pathway.^a^

DHI	Coherence				Cognitive participation				Collective action				Reflexive monitoring				Response to NPT		
	1	2	3	4	5	6	7	8	9	10	11	12	13	14	15	16	Yes	Maybe	Not reported
getUbetter	☑^b^	☑	☑	☑	⍰^c^		☑		☑	☑	⍰	☑		⍰		☑	56	19	25
Phio		⍰	☑	⍰	⍰	⍰	⍰		⍰	☑	☑		☑				25	38	38
ACL^d^ Rehab																	0	0	100
CHES^e^		⍰			☑						⍰			⍰			6	19	75
Doct’Up				⍰		⍰	⍰				⍰						0	25	75
Fitbit																	0	0	100
Khymeia system					☑					⍰	⍰					⍰	6	13	81
KNEESUP		⍰	⍰	☑	⍰					⍰	⍰						6	31	63
Limber Health DETA^f^	⍰		⍰														0	13	88
Mawendo											⍰						0	6	94
Sword Health			⍰	⍰	⍰	⍰	⍰						⍰				0	38	63
Microsoft Kinnect																	0	0	100
NeoRehab	☑	⍰	⍰									☑		⍰	⍰		13	25	63
Orthelligent			⍰	☑	⍰	⍰	⍰										6	25	69
Orthopy						⍰				⍰							0	13	88
Physitrack	☑		☑	⍰			⍰	☑					⍰				19	19	63
QoC Health Inc	⍰	☑	☑	⍰	⍰	⍰	☑				☑	⍰		⍰			25	38	38
UIN-CARE Home 1			⍰		⍰	☑					⍰			⍰		⍰	6	31	63
Joymotion			⍰		☑												6	6	88
Yes	16	11	21	16	16	5	11	5	5	11	11	11	5	0	0	5	Yes = ☑		
Maybe	11	21	37	26	37	32	26	0	5	16	37	5	11	26	5	11	Maybe = ⍰		
Not reported	74	68	42	58	47	63	63	95	89	74	53	84	84	74	95	84	Not reported=blank		

a Additional details can be found in Multimedia Appendix 1.

b The OPTIKNEE domain was provided in the content of the DHI.

c It is not clear if the OPTIKNEE domain was provided in the content of the DHI.

d ACL: anterior cruciate ligament.

e CHES: computer-based health evaluation software.

f DETA: digital exercise therapy application.

**Table 5.** Normalization process theory (NPT) results for digital health intervention (DHIs) at the evidence stage of the health technology pathway.^a^

DHI	Coherence				Cognitive participation				Collective action				Reflexive monitoring				Response to NPT		
	1	2	3	4	5	6	7	8	9	10	11	12	13	14	15	16	Yes	Maybe	Not reported
ACL-Well^b^																	0	0	100
C Anthony																	0	0	100
BANG^c^			☑^d^		⍰^e^	⍰											6	13	81
CBPT-ACLR^f^			☑	⍰	☑				⍰			☑		⍰			19	19	63
C Hadley																	0	0	100
HJT Software	⍰			⍰				⍰									0	19	81
P Levinger		⍰	⍰	⍰	☑						☑					⍰	13	25	63
L Li					⍰									⍰			0	13	88
F Litchfield	☑	☑	☑	⍰							⍰	☑		⍰			25	19	56
C Martin			☑	⍰		☑				⍰		☑					19	13	69
Nefitness							⍰				⍰						0	13	88
J Nguyen										⍰	☑						6	6	88
Rehab Cloud Platform			⍰								☑						6	6	88
SOAR^g^			⍰	⍰	⍰									⍰			0	25	75
TRAK-ACL	☑	⍰	⍰	⍰	⍰	⍰	⍰	⍰	⍰	☑	⍰	⍰		⍰	⍰	⍰	13	81	6
A Tsvyakh					⍰		⍰				☑						6	13	81
Upscaler																	0	0	100
QJ Wang																	0	0	100
B Brewer		⍰		☑	⍰												6	13	81
Yes	11	5	21	5	11	5	0	0	0	5	21	16	0	0	0	0	Yes = ☑		
Maybe	5	16	21	37	32	11	16	11	11	11	11	5	0	26	5	11	Maybe = ⍰		
Not reported	84	79	58	58	58	84	84	89	89	84	68	68	100	74	95	89	Not reported=blank		

a Additional details can be found in Multimedia Appendix 1.

b ACL: anterior cruciate ligament.

c BANG: back in the game.

d The OPTIKNEE domain was provided in the content of the DHI.

e It is not clear if the OPTIKNEE domain was provided in the content of the DHI.

f CBPT-ACLR: Cognitive behavioral–based physical therapy anterior cruciate ligament reconstruction.

g SOAR: stop osteoarthritis.

**Table 6.** Normalization process theory (NPT) results for digital health intervention (DHIs) at the development stage of the health technology pathway.^a^

DHI	Coherence				Cognitive participation				Collective action				Reflexive monitoring				Response to NPT		
	1	2	3	4	5	6	7	8	9	10	11	12	13	14	15	16	Yes	Maybe	Not reported
A Tipprom		☑^b^	☑	☑								☑		⍰^c^	⍰		31	13	56
exoRehab			⍰				⍰										0	13	88
N Kalofonos					⍰		⍰						☑	⍰		⍰	6	25	69
KC@H																	0	0	100
KneeCare	⍰	⍰	⍰	⍰	☑						☑	☑		⍰			19	31	50
G Kordatos		⍰	⍰		⍰		☑	☑	⍰		☑			⍰		⍰	19	38	44
L Wang					⍰												0	6	94
SANA	☑	⍰	☑	☑	⍰		⍰	⍰						⍰		⍰	19	38	44
SKYRE											⍰					⍰	0	13	88
Y Ling																	0	0	100
E Zadrapova											⍰						0	6	94
Yes	9	9	18	18	18	0	9	9	0	0	18	18	9	0	0	0	Yes = ☑		
Maybe	9	27	27	9	36	0	27	9	9	0	18	0	0	45	9	36	Maybe = ⍰		
Not reported	82	64	55	73	45	100	64	82	91	100	64	82	91	55	91	64	Not reported=blank		

a Additional details can be found in Multimedia Appendix 1.

b The OPTIKNEE domain was provided in the content of the DHI.

c It is not clear if the OPTIKNEE domain was provided in the content of the DHI.

DHIs were mapped against the Health-Technology Pathway (Tables 4-6), which defines the key steps in the end-to-end pathway for health technology from research to patient treatment [34]. The different stages of the health technology pathway include creation, development, evidence/regulation, commissioning, and adoption.

### Quality Assessment

Reports were assessed against the mERA (mobile health evidence reporting and assessment) checklist [35], which outlines a minimum set of information required to define what the mobile health intervention is, where it is being implemented, and how it was implemented to support replication of the intervention. It is scored on a 16-point scale with a point attributed to each item (maximum score of 16). To assess methodological quality, the Quality Assessment for Diverse Studies (QuADS) tool [36] was used, which has been used in previous scoping reviews [26,29] to assess a diverse range of studies with varied methodology. The QuADS tool has a maximum score of 39 from 13 questions, each scoring up to 3 points, with higher scores representing a higher methodological and reporting quality.

## Results

### Study Characteristics

Eighty-one reports were identified following full text screening. The final analysis includes 49 DHIs, as multiple reports referenced the same DHI (Figure 1). Study designs of the included reports were as follows: 18 randomized controlled trials (RCTs), 9 technical reports, 6 protocols, 4 qualitative reports, 4 feasibility studies, 3 mixed methods, 2 case series, 2 clinical studies, 2 retrospective observational studies, 2 quasi-experimental studies, 2 pilot studies, 2 prospective cohorts, 2 retrospective cohorts, 2 secondary analyses of a prospective cohort study, 2 service evaluations, and one app review, case report, case-control study, pilot RCT, superiority trial, registry study, repeated measures design, retrospective study, usability study, validation study, service development project, qualitative mapping study, Delphi study, mixed methods service evaluation, case study, quality improvement project, feasibility and usability study, secondary analysis of an observational study, and a cost-effectiveness analysis (Multimedia Appendix 1). The highest score on the mERA checklist was 10 out of 16 (mean=4.75, SD 2.12), and the highest score on the QuADS assessment tool was 36 out of 39 (mean=20.19, SD 8.87).

**Figure 1.**
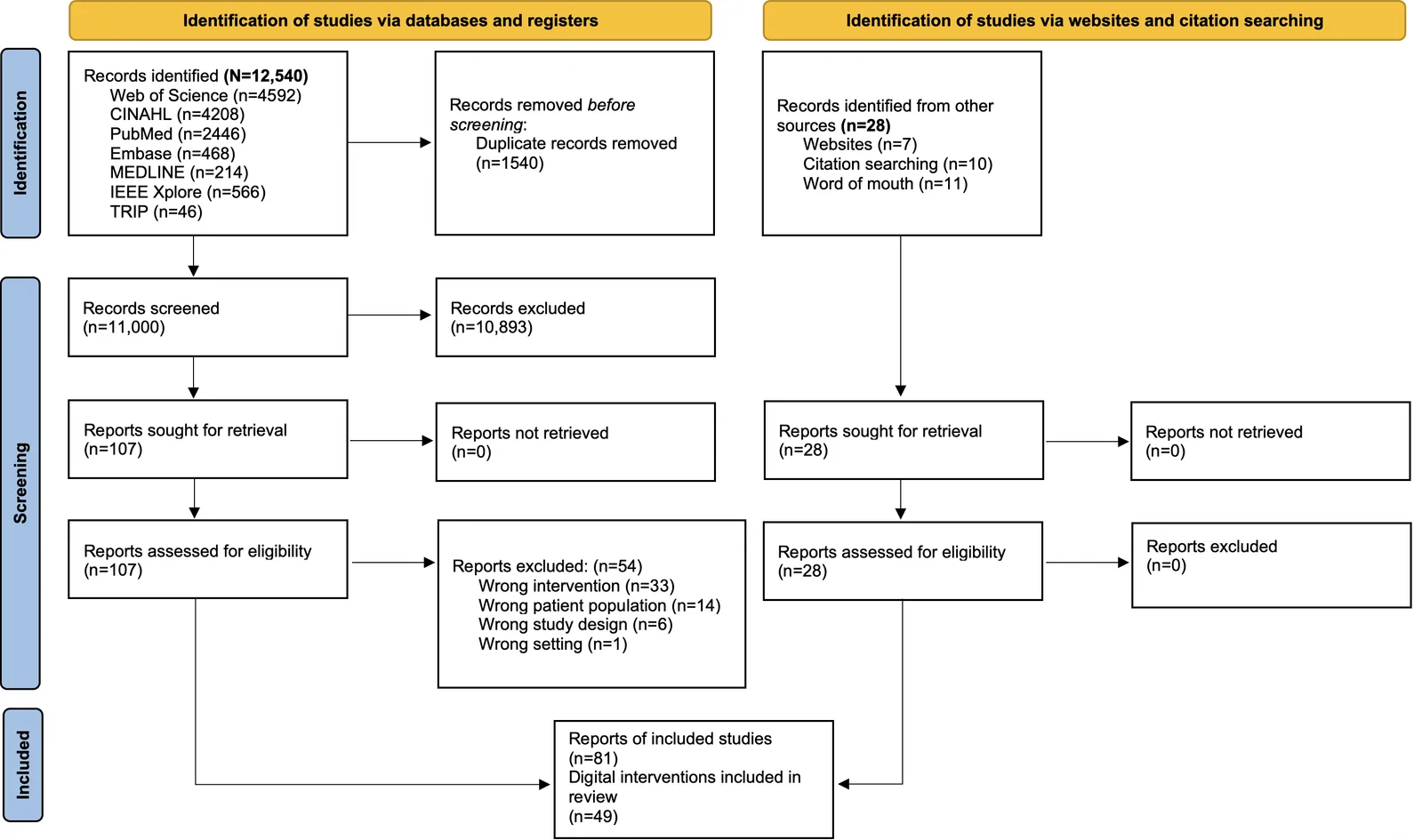
PRISMA diagram. PRISMA: Preferred Reporting Items for Systematic Reviews and Meta-Analyses.

Twelve DHIs focused on managing isolated ACL ruptures, 15 for ACL rupture ± meniscus involvement, 3 for acute meniscus tears, 2 ACL rupture or meniscus tear, 2 knee fractures, and 1 patella dislocation. 14 were not specific to a type of knee injury, but the DHI had the potential to be used with patients who had sustained a TKI. Across the 49 different DHIs, 25 were classified as mobile health, 19 as telerehabilitation, 4 as eHealth, and one as both telerehabilitation and mobile health. There were 19 DHIs at the market stage, 19 were at the evidence stage, and 11 were at the development stage (full list of references with corresponding characteristics in the Multimedia Appendix 1).

### Mapping DHIs Against OPTIKNEE

The highest OPTIKNEE score from a DHI was 4 out of 7 (57%), and exercise provision was the most reported recommendation at 41 (84%). Information provision scored 37%, PROMS scored 35%, goal setting scored 22%, and overall patient outcome scored 24%. There was only one self-assessed functional assessment reported, and no self-assessed strength assessments were reported. Eight DHIs reported 4out of 7 (57%) of the OPTIKNEE recommendations as part of their content; however, 18 DHIs scored 1 out of 7 (14%) and 2 out of 7 (28%). There were 36 out of 49 (73%) DHIs that had 2 or fewer of the OPTIKNEE recommendations included in their content.

### NPT Results

The NPT results have been broken down into 3 tables to illustrate the stage each DHI is at on the health-technology pathway, and key quotes can be seen in Figure 2. The health-technology stages include market, evidence, and development. A green box indicates a “positive” response to one of the 16 questions. Yellow indicates “maybe,” and gray means that it was “not reported” (see Multimedia Appendix 1 for the full set of NPT questions).

**Figure 2.**
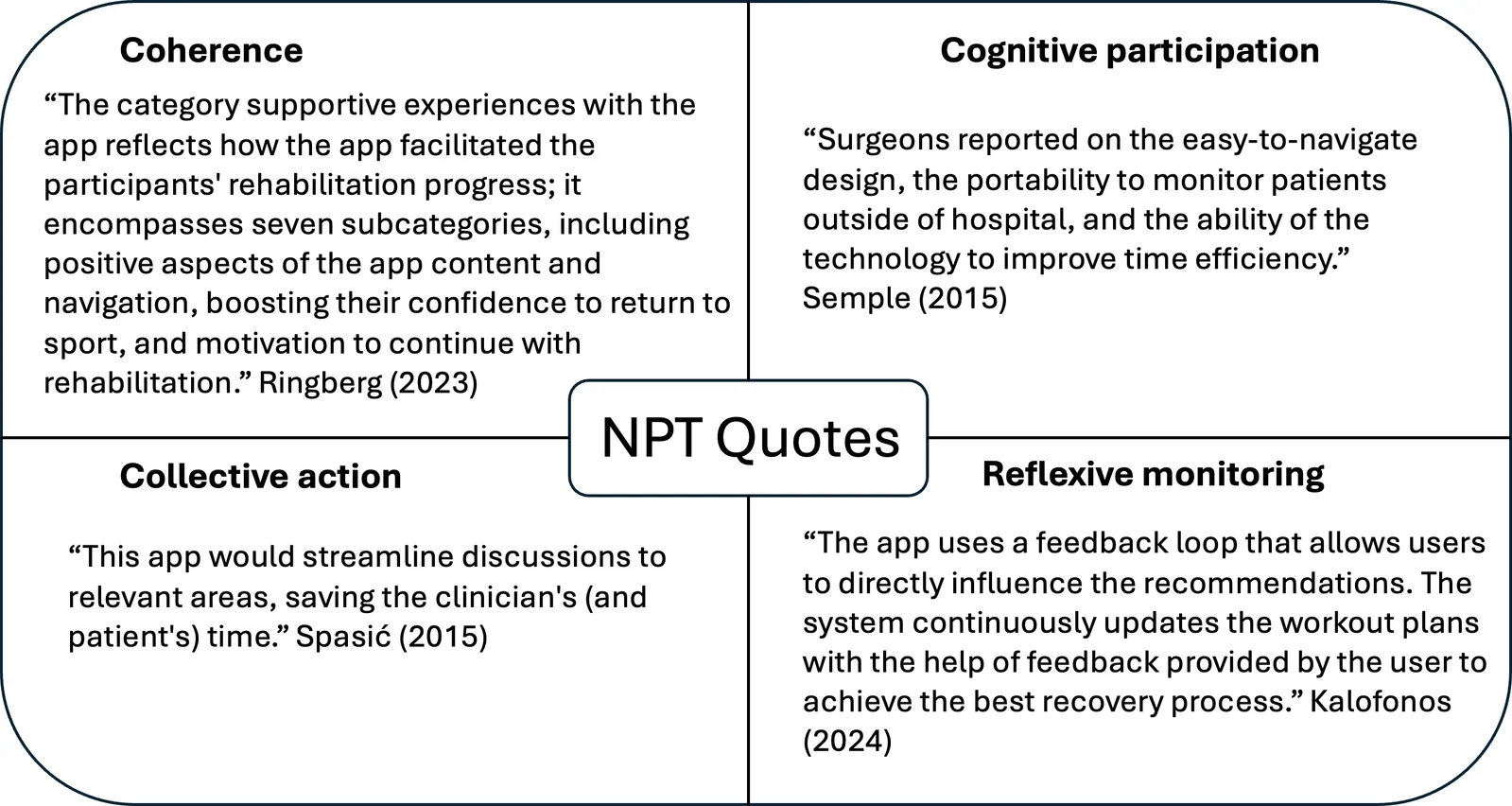
Key normalization process theory (NPT) quotes from the included reports for each of the NPT domains (Multimedia Appendix 1 for a full set of quotes for each NPT question) [37-42].

At the market stage, the DHI with the highest positive responses was “getUbetter” at 9 (56%). Within the evidence stage, the highest positive response for a DHI was F. Litchfield, at 25% (n=4). Then the highest positive response across the NPT questions was 21% (n=10) for the third and eleventh questions. For DHIs in the development stage, A. Tipprom scored the highest positive response at 31% (n=5). However, the highest positive response for the NPT questions was 18% (n=2) for the third, fourth, fifth, eleventh, and twelfth questions. Across all stages of the health-technology pathway, the most common positive score for NPT was 0.

For the health-technology pathway, out of 49 DHIs, 39% (n=19) were at the market stage, 39% (n=19) at the evidence stage, and 22% (n=11) in the development stage. Despite being in the market stage, there were a total of 28 out of 304 positive responses across the 4 domains, while DHIs at the evidence stage and at the development stage showed 19 out of 304 and 15 out of 176 positive responses, respectively. This lack of noticeable differences between stages seems to also be present when considering individual NPT domains.

## Discussion

### Overview

In addressing our aim to evaluate DHIs against the OPTIKNEE consensus guidelines [7], we found that all identified DHIs lack the required content and features to promote knee health in people at high risk of PTOAK after a TKI. Although the DHIs have been variably adopted (19 on the market, 19 with evidence, and 11 in development), none demonstrate evidence of having reached the required criteria or spread to be regarded as accepted in practice. It is difficult to pinpoint exactly why there is variable DHI adoption from the evidence, and it is likely multifactorial.

### Digital Adoption

The NPT framework was applied to the DHIs to explore if and to what extent these DHIs were adopted into routine practice. The NPT framework has been applied in previous research such as a scoping review [43] and in qualitative research [44,45]. It is possible for DHIs to be implemented into practice successfully, and a previous example of this was the HeLP-Diabetes web-based self-management program, which was effective at helping people with type 2 diabetes to support their glycemic control [46]. If a DHI is not adopted, patients may have less successful outcomes [47], and it may lead to lost opportunities to address health inequalities [48]. A previous scoping review found that there are no clear implementation strategies that are effective for the adoption of DHIs in rehabilitation [49].

Results from the NPT analysis in this review demonstrate some successful adoption of PTOAK-relevant DHIs into routine practice. GetUBetter had positive responses in over half of NPT questions, and Phio and QoC Health Inc had positive responses in over a quarter of NPT questions. In this review, DHIs are aimed at patients, clinicians, or both, which may be why the positive responses in the NPT analysis were variable. Previous evidence found that perceived effectiveness was key for clinicians adopting a new DHI [50]. To enhance engagement with patients, the DHI needs to be low effort and meet the specific needs of the patient [51]. As the NPT analysis is an accurate measure of adoption, then we would expect there to be higher levels of adoption for DHIs on the market compared to DHIs in development. However, our results illustrate that there was no clear pattern between the DHI stage of development and adoption into routine practice (Tables 4-6). This would suggest that there is insufficient development prior to DHIs being implemented into routine practice, possibly explaining the lack of functionality and guideline compliance. Information typically presented in different study designs might justify the variability in scoring of the NPT framework. For example, RCTs are unlikely to report whether individuals have a clear understanding of the DHI in a trial report but may do in a process evaluation. The lack of clear, comprehensive reports of guideline-compliant intervention development perhaps explains the lack of efficacy studies.

### OPTIKNEE Digital Delivery

When DHI content was mapped against OPTIKNEE [7] guidelines, the exercise domain scored the most positive results (84%), recommended as a core treatment for knee injuries and PTOAK prevention [7]. Exercise content across the DHIs was mixed depending on the target audience, as some DHIs are targeted at a more general population compared to those with specific knee injuries. This means that exercises provided to patients through some DHIs do not address the specific rehabilitation needs of a patient post TKI. This would suggest that the DHIs not providing appropriate knee-focused exercises may put patients at an increased risk of developing PTOAK after a TKI.

Information provision was present in only 37% of DHIs, highlighting that education and information provision are rarely being provided despite it being widely recommended as a first-line treatment for most musculoskeletal conditions [52]. Education is vital to help patients address and avoid risk factors such as weight gain or inactivity [7]. Although OPTIKNEE refers to this recommendation as “education,” we decided to use the term “information provision” instead as some DHIs only had basic information on the condition rather than formal education strategies, with testing of learning. This review highlights that most DHIs were not addressing this OPTIKNEE recommendation [7], therefore reducing the likelihood of preventing PTOAK.

PROMS were integrated into 35% of DHIs as a method of data collection and represent a validated method of establishing progress or health care outcomes [53,54], being relatively easy and efficient. It could also be a way of monitoring patients in the long term and flagging when they may be at risk of functional decline. OPTIKNEE recommendations [7] provide clear guidance on which PROMS should be used in this patient population; this includes the IKDC-SKF [55], KOOS [56], and WOMET [57]. However, there are a wide selection of different PROMS identified in the DHI included in this review, meaning guideline compliance is poor and efforts to compare efficacy are hindered.

The OPTIKNEE guidance [7] recommends in-person functional movement assessments, such as a crossover hop test, as a key performance marker to monitor knee-related function in people at risk of PTOAK. Only Orthelligent contained a digitally supported self-assessed functional assessment, in this case a vertical hop test. It may not be the gold standard, but remote self-assessed functional tests can be completed via DHIs. To assess children’s physical fitness, a DHI was developed to assess coordination, muscular endurance, and flexibility, which was found to be valid and reliable [58]. Remote smartphone-based balance assessments have been completed successfully for people with Parkinson disease [59], and vertical jumps, a common and important test, have been demonstrated as valid and reliable in a digital format [60]. By taking a pragmatic approach to assessing functional movements via a DHI, it will allow clinicians to effectively monitor these patients at risk of developing PTOAK after a TKI.

For strength assessments, the OPTIKNEE guidance [7] recommends an in-person strength assessment completed via a one-rep max test or dynamometry. It may not be an in-person strength assessment as recommended by OPTIKNEE [7], but no DHI reported a self-assessed strength test. If thigh muscle strength is not maintained, it can put the patient at an increased risk of PTOAK [7]. Similar to functional assessments, there are mobile apps that can remotely assess strength [61], and other strength tests could be incorporated digitally such as the single-leg wall squat test [62]. This is difficult to replicate via a digital intervention, but a remote strength test could supplement gold standard strength testing that is usually completed in the clinic environment (ie, better to monitor strength in some format rather than not at all). By monitoring strength remotely, it would provide another avenue for clinicians to track patients who are at risk of developing PTOAK.

Mixed reality and immersive virtual reality could support remote musculoskeletal care by facilitating assessments and supporting remote exercise provision [63,64]. These tools can provide biofeedback for patients and support gait retraining during physical rehabilitation [64]. Mixed or immersive virtual reality could also address the difficulty of performing remote strength and functional assessments by, for example, using virtual reality to guide patients through a hop test or a squat assessment remotely. Immersive virtual reality has already been successful in providing rehabilitation to improve function to patients after a traumatic hand injury [63]. Though these options show potential, they would need to be integrated into current clinical pathways to ensure they can be adopted into routine practice. However, limitations regarding initial upfront cost, time spent onboarding patients, and limited engagement from individuals with reduced digital literacy skills will also need to be addressed when using these systems.

Clinical outcomes were highlighted in 24% of DHIs. It included metrics such as knee-related pain, QoL, adverse events, and physical activity levels. Collecting these metrics could be easily delivered via a DHI, and there is evidence that some patients prefer this over paper-based approaches [65]. Goal setting was present in 22% of DHIs; however, previous evidence has demonstrated that goal setting can be delivered via a DHI and is widely accepted by patients [66]. This is vital to promote patients’ engagement and support patients after a TKI, leading to improved patient outcomes and knee-related QoL by reducing fear and anxiety [7].

AI agents or large language model (LLM)–based assistants are another option for supporting digital musculoskeletal care. They could provide clinical decision support and integrate with current workflows [67] and provide health education [68] and basic rehabilitation planning [69]. These models could be used to facilitate OPTIKNEE recommendations, informing a DHI to deliver information provision, goal setting, outcome monitoring, and basic rehabilitation provision without fragmentation of care to patients. Clinically validated agents are sparse despite this area developing rapidly; however, current models are limited by a lack of interpersonal connection, public distrust in AI, and ethical concerns regarding dataset bias. Those would need to be addressed prior to implementation into musculoskeletal care.

Although all DHIs included could be used to reduce the risk of PTOAK, most did not specifically state this as an aim. The DHI by Martin et al [70] was the one exception, and the study was published after the release of the OPTIKNEE recommendations. The DHI reported is explicitly focusing on reducing the risk of PTOAK after a TKI in military personnel. However, despite aiming to mitigate the risk of PTOAK, it still lacked some of the components outlined by the OPTIKNEE consensus: strength and functional assessments [7]. These may not have been considered by the researchers, may have been too difficult to build into the DHI, or may have felt it was not required as military personnel are routinely tested for strength and functional performance in their occupation [71]. However, the results from this preliminary RCT demonstrate that participants in the DHI group had better pain control strategies and fatigue management [70].

At present, there is no single DHI that can provide all the OPTIKNEE [7] recommendations to patients after they have sustained a TKI. The results highlight the variability of what is offered by different DHIs targeted at supporting individuals post TKI. The results demonstrate a lack of standardized digital care being provided to patients after TKI, despite there being clear guidance to reduce the risk of these patients developing PTOAK. This indicates that there is a gap in service provision to support patients after TKI and reduce their risk of developing PTOAK. Future services will need integrated digital solutions to ensure care priorities are met, that is, analog to digital, and outcomes optimized.

### Implications

A future evidence-informed digital intervention that can incorporate all aspects of the OPTIKNEE recommendations [7] in a pragmatic manner would be a valuable intervention to support patients after a TKI to reduce their risk of PTOAK. Key features of successful DHIs implemented into routine practice have demonstrable clinical benefit [72], ability to scale the digital intervention [73], can be easily integrated into routine care [74], and there is perceived value from clinicians [75]. However, when developing new DHIs, digital literacy needs to be considered because it remains a barrier to patients engaging with DHIs, and future digital innovations should provide equitable health care [76]. A future DHI would require all these features to ensure that it is successfully implemented into routine practice. Due to the complex nature of TKIs and varying levels of severity, there would need to be future research about when is best to implement a DHI in the care pathway for patients. Additionally, a user-centric design during development is an essential strategy for the successful implementation of DHIs, as input from the TKI population during the development can lead to greater acceptance and perceived usefulness of the DHI and consequently improved engagement and outcomes [77]. It would also be important to explore which clinicians are best placed to deliver and onboard patients to a DHI after a TKI.

### Strengths and Limitations

This review was able to include a wide variety of different DHIs by adopting a broad, extensive, and creative search strategy, including academic and nonacademic sources, resulting in an analysis that can provide a clear picture of the DHIs being used with this patient population. The review methods were adapted due to the nature of the topic while still being reproducible and applying clear frameworks for data analysis. Although we included a wide search strategy, most of the reports in the review were designed for an ACL-injured population, and there was a paucity of evidence on other TKIs. It is possible that DHIs are being more widely adopted than our review shows. Although the NPT framework has been shown as useful in similar contexts, its results are dependent on the quality and volume of the reports, which may not reflect what is occurring in routine practice, with few DHIs undergoing thorough formal intervention development.

### Conclusion

This scoping review identified 49 DHIs being used with or having the potential to be used with individuals post TKI. The DHIs are at various stages of development in the health technology pathway, which is not necessarily associated with the level of adoption. Ultimately, most DHIs lack self-assessed functional and strength assessments, indicating that rigor is insufficient across the DHIs. This review did not identify a DHI that can provide all the OPTIKNEE recommendations, which aims to minimize the risk of patients developing PTOAK in the long term. Future research should focus on measuring the efficacy and feasibility of an OPTIKNEE-informed DHI to support individuals after TKI.

## Supplementary material

10.2196/99607 Multimedia Appendix 1 Additional information regarding search strategy, inclusion/exclusion criteria, normalization process theory questions, and quotes.

10.2196/99607 Checklist 1 PRISMA checklist.

## Abbreviations

DHI: digital health intervention
FDA: Food and Drug Administration
LLM: large language model
mERA: mobile health (mHealth) evidence reporting and assessment checklist
MHRA: Medicines and Healthcare products Regulatory Agency
NPT: normalization process theory
PRISMA-ScR: Preferred Reporting Items for Systematic Reviews and Meta-Analyses Extension for Scoping Reviews
PROM: patient-reported outcome measure
PTOAK: posttraumatic osteoarthritis knee
QoL: quality of life
QuADS: quality assessment with diverse studies
RCT: randomized controlled trial
TKI: traumatic knee injury
