# Supplementary material for: Digital Health Interventions to Prevent Posttraumatic Arthritis After Traumatic Knee Injury: Scoping Review
Source: J Med Internet Res. 2026 Oct 28;28:e99607. doi: 10.2196/99607 (PMC13732755; doi:10.2196/99607)
Supplement: Supplementary file 2 [file jmir-v28-e99607-s001.doc]

**Appendix: Search Strategy**

For this scoping review it was necessary to use a non-standard search strategy to make sure that reports including digital health interventions were captured outside of research studies. Academic databases were searched initially to capture published reports. Grey literature was also searched to find potential digital interventions that may have been presented at academic conferences but not formally written up as a research publication. Details of the searches can be found in the methods section of the main text and they followed a standardised approach. Searches were imported into the software known as Covidence to facilitate a title / abstract screen followed by a full text screen. The screening process was completed in full by JC and a second round of screening at all stages was completed by RR and two other contributors who have not been listed as authors. Other sources included relevant websites such as: imedicalapps.com, Innovate UK, NHS Digital Playbook, Medicines and Health Regulation Agency (MHRA), Food and Drug Administration (FDA), Google Scholar, Queen Mary Research Online, Versus Arthritis and the University of British Columbia Open Collections. When searching other sources a pragmatic approach was employed, if there was a high volume of results for example in the thousands then the first 100 results were screened for relevant reports. The lead author also completed a search of citations from relevant review articles that appeared in the initial searches of academic databases.

**Search term:**

'knee dislocation’ OR 'acl' OR ‘anterior cruciate ligament’ OR 'meniscus' OR 'knee fracture' OR ‘knee injury’

**AND**

'wearable device' OR 'mobile application' OR ‘mobile applications’ OR ‘mobile apps’ OR ‘mobile app’ OR 'internet' OR 'mobile phone' OR ‘smart phone’ OR 'telemedicine' OR 'technology' OR 'mhealth' OR 'telehealth' OR ‘emedicine’ OR ‘mobile health’ OR ‘cell phone’ OR 'digital health technology' OR 'digital health'

**AND**

'outcome assessment' OR 'monitoring' OR 'kinesiotherapy' OR 'signposting' OR 'advice' OR 'education' OR 'telerehabilitation' OR 'exercise' OR 'rehabilitation' OR 'patient reported outcome measure' OR ‘diet’

Google Scholar

A search of google scholar was completed using the main search term described above. This returned over 150,000 results, however only the first 100 results were screened. There were no relevant reports identified.

iMedicalApps.com

iMedicalApps is an online publication for medical professionals, patients and analysts interested in mobile medical technology and health care apps. It includes a team of physicians, allied health professionals, medical trainees and mhealth analysts that provide reviews, research and commentary of mobile health technology. After a manual search of the sports medicine, orthopaedic and physical therapy section, there was one result.

Table S1. Example of the digital health intervention considered for analysis

| **Name** | **Reason for inclusion** |
| --- | --- |
| ACL Rehab | Focused on a traumatic knee injury and contains information provision. |

Innovate UK

Innovate UK is part of UK research and Innovation and it serves to help commercialise research into real world applications. The innovate UK database has over 60,000 projects listed which meant that it was not feasible to examine them all in detail due to a small research team. A search of the projects was completed using the terms “digital” AND “knee” which yielded 1 possible result.

Table S2. Example of the digital health intervention considered for analysis

| **Name** | **Accept/ Reject** | **Exclusion reason** |
| --- | --- | --- |
| MuJo Mechanics Ltd | Reject | Not related to knee injuries |

NHS Digital Playbook

The NHS digital playbook provides real world examples of digital innovations that have been implemented into practice to inspire future innovators. For this a search of the musculoskeletal (MSK) section was completed.

Table S3. Example of the digital health intervention considered for analysis

| **Name** | **Reason for inclusion** |
| --- | --- |
| GetUBetter | Has the potential to be used with people after a traumatic knee injury |

Medicines and Health Regulation Agency

A search of the medicines and health regulation agency (MHRA) website was completed, because this database contains a list of approved applications. The result only yielded two results which were not relevant. The search term used was “digital health” AND “knee injury”. A problem with this website is that you can only search using the product name, this may have led to limited results.

Food and Drug Administration

A search of the food and drug administration (FDA) database was completed using the following search terms “digital health” AND “knee injury”. The results yielded 654 results and after individually screening there were no results which were deemed relevant for the final analysis.

Queen Mary Research Online

Queen Mary Research Online (QMRO) is an open access institutional repository of research output which has been undertaken at Queen Mary University of London. A search of this website was completed using the following search terms “digital health” AND “knee injury”. This produced 941 results and the lead author evaluated the first 100 results, but there were not any relevant reports included in the final analysis.

Arthritis UK (Previously Versus Arthritis)

Arthritis UK is a charity focused on creating lasting change for people living with arthritis. A key focus of the charity is around the prevention of arthritis which is also a focus of this review hence why this website was search to see if there were any relevant digital health interventions. The following search terms were used “digital health” AND “knee injury” which produced 130 results however no results were deemed relevant for the final analysis.

University of British Columbia Open Collections

The University of British Columbia Open Collections includes a publicly accessible collection of resources which reflect the research interests of the University of British Columbia community. The website was searched using the search terms “digital health” AND “knee injury” and it produced 783 results. The first 100 results were evaluated but zero were relevant for the final analysis.

**Inclusion/ Exclusion Criteria**

A digital health intervention was defined as a health intervention delivered through digital tools or communication technologies which collect, store, share, and analyse health information for the purpose of improving patient health and care, this could be through wearable devices, mobile apps, text with smartphones or telehealth. For this review we kept the inclusion criteria broad to try and ensure that we captured a plethora of different digital health interventions aimed at supporting individuals after a traumatic knee injury.

No preference was given to any system regarding their stage of development. This meant that digital health interventions were included if they met the inclusion criteria and they could be at any stage of development from protypes to those already on the market. Digital health interventions were also included in the review if they had the potential to be used with people who have sustained a traumatic knee injury such as technical papers or wearables.

There were three digital health intervention that met the inclusion criteria initially but were not included due to a lack of available information about the intervention and its implementation. For example Recoveryone met the inclusion criteria but after a search of the literature, company website and the internet using the name of the digital intervention but we could not find enough information to conduct the analysis. Kaia Health was initially included but then after reviewing the literature the only available evidence for the DHI was focused on treating low back pain so did not meet the inclusion criteria. Hinge Health was initially included but on further investigation it was found to be a DHI that specifically focuses on helping people who have chronic pain so did not meet the inclusion criteria.

The full inclusion and exclusion criteria can be found below and in the main text.

Table S4. Inclusion and exclusion criteria

| **Inclusion** | **Exclusion** |
| --- | --- |
| - Reports which include a DHI (this includes but is not limited to telemedicine, mhealth, telerehabilitation, mobile application, telehealth or wearable device including virtual reality). - Reports which include patients who have had a TKI. Including but not limited to ACL rupture (pre or post operation), meniscus tear, intra-articular tibiofemoral fracture or patella dislocation. - Reports including at least one of: digitally supported outcome measures, weight management strategies, physical monitoring (including wearables), exercise prescription, signposting or education. | - Reports which do not include a TKI - Reports which do not include a DHI that could be used with people who have had a TKI. - Reports which included participants with osteoarthritis or participants who have undergone joint arthroplasty. - Reports not reporting one of the following: outcome measures, weight management strategies, physical monitoring (wearables), exercise prescription, signposting or information provision/ education. - Reports which involve the use of exoskeletons or EMG. |

Table S5. The NPT domains and the individual questions

| Domains | NPT Question |
| --- | --- |
| Coherence – the participants | Q1. Is there a clear understanding about the difference between the digital health intervention and a non-digital approach? |
|  | Q2. Do individuals have a shared understanding of the digital health tool or intervention? |
|  | Q3. Do individuals have a subjective understanding about the digital health tool or intervention? |
|  | Q4. Do individuals understand the potential value and benefit of the intervention? |
| Cognitive Participation – engagement from the participants | Q5. Do individuals "buy in" to the new digital health intervention? |
|  | Q6. Can individuals sustain the use of the digital health intervention? |
|  | Q7. Are key individuals willing to drive the implementation of the digital intervention? |
|  | Q8. Do individuals believe it is right for them to be involved in the use of the digital intervention? |
| Collective Action – the participants working to make the intervention function | Q9. How does the digital intervention affect the roles and responsibilities or training needs of the staff involved? |
|  | Q10. Is there organizational or company support? |
|  | Q11. Does the digital intervention make people's work easier? |
|  | Q12. Do individuals have confidence in the digital health intervention? |
| Reflexive Monitoring – the participants reflecting on or appraising the intervention | Q13. Do individuals try to alter the use of the digital health intervention? |
|  | Q14. How do groups judge the value of the digital health intervention? |
|  | Q15. How do individuals appraise the effects of the digital health intervention on them and their work environment? |
|  | Q16. How are benefits or problems identified or measured? |

**NPT domain quotes**

Q1)

- “A high number of participants expressed a preference for traditional, face-to face assessment if given the choice, however they were very supportive of telerehabilitation processes if remote consultations were the only option.” Richardson (2017) [1]
- "Patients were very clear that they did not think TRAK was a replacement for physiotherapy but should be used to complement face-to-face treatment in a 'blended approach'." Button (2015) [2]

Q2)

- "Participants described their satisfaction with the device as excellent (43%), good (40%), fair (10%), and poor (7%), and 94% of patients reported that they would respond to questions using a similar application in the future. Both physicians rated their experience as positive and identified useful traits in the web portal." Higgins 2017 [3]

Q3)

- "Experience questionnaires revealed the majority found the video easy to access and understand, met expectations and would use something similar again." Litchfield (2019) [4]
- "The category Supportive experiences with the app reflects how the app facilitated the participants' rehabilitation progress; it encompasses seven subcategories, including positive aspects of the app content and navigation, boosting their confidence to return to sport, and motivation to continue with rehabilitation." Ringberg (2023) [5]

Q4)

- "The post experimental questionnaire results attest to the acceptability of the interactive multimedia program to participants. The multimedia program was rated as more useful than standard care for an array of tasks pertaining to coping with the rigors of postoperative rehabilitation and the return to sport." Brewer (2022) [6]
- "Some participants preferred telerehabilitation because of the decreased number of required hospital visits during the COVID-19 pandemic and the fact that daily rehabilitation could be monitored directly by us through a web portal during the postoperative period." WJ Liao (2022) [7].

Q5)

- "86.9% of patients were satisfied or very satisfied with the whole process of electronic PRO assessment over the 12 months period." Olach (2021) [8].
- "Participants acknowledged that the Mobile MM was advantageous for addressing their psychological barriers after ACLR as observed by self-reported changes in fear and confidence on the acceptability questionnaire." Baez (2023) [9]

Q6)

- "The Mobile MM intervention demonstrated excellent retention and adherence and was moderately to highly acceptable to participants." Baez (2023) [9]
- "According to the Transtheoretical Model, the "maintenance phase" of change indicates that participants have adopted the selected behaviour and are working to sustain the behaviour long term (i.e., >6 months). The treatment group reported an increase in maintenance for pain control strategies and fatigue management, which may have contributed to the differences in constant knee pain reported at the 6-week follow-up." Martin (2024) [10]

Q7)

- “Surgeons reported on the easy-to-navigate design, the portability to monitor patients outside of hospital, and the ability of the technology to improve time efficiency." Semple (2015) [11]

Q8)

- "Patients expressed a willingness to engage in a hybrid model with early in-person rehabilitation followed by later telerehabilitation, which may result in more optimal outcomes." Vasavada (2024) [12]

Q9)

- "Clinicians felt that the barriers could be overcome by offering more training for the clinicians as well as the administrative staff on the capabilities of the app, which would allow them to help guide patients and provide reassurance around the safety netting mechanisms of the app." Wanless (2024) [13]

Q10)

- "Physiotherapists discussed the risks associated with TRAK training and management for physiotherapists. Physiotherapists highlighted organisational changes are needed to better integrate its use into standard physiotherapy practice." Button (2017) [2]

Q11)

- "Participants from the intervention group rated the internet based intervention as a useful tool for reinforcing rehabilitation exercises and the SMS reminders to complete their physiotherapy exercise program as important." Levinger (2017) [14]
- “This app would streamline discussions to relevant areas, saving the clinician's (and patient's) time.” Spasić (2015) [15]

Q12)

- “When asked about how helpful CBPT-ACLR was to recovery, patients reported a mean score of 7.6 from 5 to 10, indicating moderate to high degree of helpfulness.” Coronado (2020) [16]

Q13)

- "The app uses a feedback loop that allows users to directly influence the recommendations. The system continuously updates the workout plans with the help of feedback provided by the user to achieve the best recovery process." Kalofonos (2024) [17]

Q14) No positive responses

Q15) No positive responses

Q16)

- "A questionnaire was developed to assess the 'acceptability' of getUBetter for patients and clinicians. Group NU answered the following questions with answers highlighting potential digital inclusion issues: Q16: If you have found the app difficult to use or unhelpful please tell us why - 24 people cited reasons around digital inclusion. These mainly focused on understanding the information within the app and difficulty in using and navigating the app." Wanless (2024) [13]

**NPT Quote References:**

1.	Richardson BR, Truter P, Blumke R, Russell TG. Physiotherapy assessment and diagnosis of musculoskeletal disorders of the knee via telerehabilitation. J Telemed Telecare 2017 Jan 8;23(1):88–95. doi: 10.1177/1357633X15627237

2.	Dunphy E, Hamilton FL, Spasić I, Button K. Acceptability of a digital health intervention alongside physiotherapy to support patients following anterior cruciate ligament reconstruction. BMC Musculoskelet Disord BioMed Central Ltd.; 2017 Nov 21;18(1). PMID:29162071

3.	Higgins J, Semple J, Murnaghan L, Sharpe S, Theodoropoulos J. Mobile Web-Based Follow-up for Postoperative ACL Reconstruction: A Single-Center Experience. Orthop J Sports Med 2017 Dec 1;5(12). doi: 10.1177/2325967117745278

4.	Litchfield F, Buttress S. Internet educational videos: improving functional recovery following arthroscopic meniscectomy? Physiotherapy 2019 Jan;105:e93–e94. doi: 10.1016/j.physio.2018.11.067

5.	Ringberg M, Eldh AC, Ardern CL, Kvist J. Athletes’ experiences of using a self-directed psychological support, the BAck iN the Game (BANG) smartphone application, during rehabilitation for return to sports following anterior cruciate ligament reconstruction. BMC Sports Sci Med Rehabil BioMed Central Ltd; 2023 Dec 1;15(1). doi: 10.1186/s13102-023-00731-2

6.	Brewer BW, Van Raalte JL, Cornelius AE. An interactive cognitive-behavioural multimedia program favourably affects pain and kinesiophobia during rehabilitation after anterior cruciate ligament surgery: an effectiveness trial. Int J Sport Exerc Psychol 2022 Jul 4;20(4):1133–1155. doi: 10.1080/1612197X.2021.1934712

7.	Liao W-J, Lee K-T, Chiang L-Y, Liang C-H, Chen C-P. Postoperative Rehabilitation after Anterior Cruciate Ligament Reconstruction through Telerehabilitation with Artificial Intelligence Brace during COVID-19 Pandemic. J Clin Med 2023 Jul 24;12(14):4865. doi: 10.3390/jcm12144865

8.	Olach M, Meester J, Behrens G, Giesinger J, Badulescu M, Giesinger K. Feasibility of Web-Based Patient-Reported Outcome Assessment After Arthroscopic Knee Surgery: The Patients’ Perspective. 2021.

9.	Baez S, Genoese F, Reiche E, Harkey M, Kuenze C, Moser J, Pietrosimone B. Feasibility of Mobile Application-Delivered Mindfulness Meditation for Individuals After Anterior Cruciate Ligament Reconstruction. International Journal of Athletic Therapy and Training 2024 Mar 1;29(2):95–100. doi: 10.1123/ijatt.2023-0004

10.	Martin CL, Nocera M, Mercer J, Marshall SW, Davi SM, Curtin JJ, Cameron KL. Efficacy of a Novel Telehealth Application in Health Behavior Modification and Symptomology in Military Service Members at Risk for Post-traumatic Osteoarthritis. Mil Med Oxford University Press; 2024 Sep 1;189(9–10):2060–2068. PMID:37966139

11.	Semple JL, Sharpe S, Murnaghan ML, Theodoropoulos J, Metcalfe KA. Using a Mobile App for Monitoring Post-Operative Quality of Recovery of Patients at Home: A Feasibility Study. JMIR Mhealth Uhealth 2015 Feb 12;3(1):e18. doi: 10.2196/mhealth.3929

12.	Vasavada KD, Shankar DS, Avila A, Mojica ES, Hurley ET, Lehane K, Buzin SD, Oeding JF, Stein SM, Gonzalez-Lomas G, Alaia MJ, Strauss EJ, Jazrawi LM, Campbell KA. Severe Attrition and Poor Satisfaction in Patients Undergoing Telerehabilitation vs. Standard In-Person Rehabilitation after Arthroscopic Rotator Cuff Repairs and Anterior Cruciate Ligament Reconstructions. Surgeries 2024 Aug 8;5(3):627–639. doi: 10.3390/surgeries5030050

13.	Wanless B, Hassan N, McClellan C, Sothinathan C, Abelleyra Lastoria DA, Herweijer T, Thorpe J, Noblet T, Berry A. How Do We Better Serve Excluded Populations When Delivering Digital Health Technology? Inclusion Evaluation of a Digital Musculoskeletal Self‐Management Solution. Musculoskeletal Care 2024 Sep 23;22(3). doi: 10.1002/msc.1925

14.	Levinger P, Hallam K, Fraser D, Pile R, Ardern C, Moreira B, Talbot S. A novel web-support intervention to promote recovery following Anterior Cruciate Ligament reconstruction: A pilot randomised controlled trial. Physical Therapy in Sport Churchill Livingstone; 2017 Sep 1;27:29–37. PMID:28822956

15.	Spasić I, Button K, Divoli A, Gupta S, Pataky T, Pizzocaro D, Preece A, van Deursen R, Wilson C. TRAK App Suite: A Web-Based Intervention for Delivering Standard Care for the Rehabilitation of Knee Conditions. JMIR Res Protoc 2015 Oct 16;4(4):e122. doi: 10.2196/resprot.4091

16.	Coronado RA, Sterling EK, Fenster DE, Bird ML, Heritage AJ, Woosley VL, Burston AM, Henry AL, Huston LJ, Vanston SW, Cox CL, Sullivan JP, Wegener ST, Spindler KP, Archer KR. Cognitive-behavioral-based physical therapy to enhance return to sport after anterior cruciate ligament reconstruction: An open pilot study. Physical Therapy in Sport 2020 Mar;42:82–90. doi: 10.1016/j.ptsp.2020.01.004

17.	Kalofonos N, Troussas C, Krouska A, Mylonas P, Sgouropoulou C. A Personalized Fitness Application for Injury Rehabilitation Using Artificial Neural Networks. 2024 19th International Workshop on Semantic and Social Media Adaptation &amp; Personalization (SMAP) IEEE; 2024. p. 56–62. doi: 10.1109/SMAP63474.2024.00020

Table S6. Reference list of included reports

| **Reference** | **DHI** | **Injury** | **Classification** |
| --- | --- | --- | --- |
| Alegrete J, Batalha N, Fernandes O, Parraca JA, Rodrigues AM, Londral AR, Sousa JP. Effectiveness of the KC@ H programme compared with clinic-based rehabilitation in patients recovering from ACL reconstruction: a study protocol for a single-centre, two-arm, single-blinded, randomised controlled superiority trial. BMJ Open Sport & Exercise Medicine. 2024 Feb 21;10(1). | KC@H | ACL rupture | Telerehabilitation |
| Anthony CA, Rojas EO, Keffala V, Glass NA, Shah AS, Miller BJ, Hogue M, Willey MC, Karam M, Marsh JL. Acceptance and commitment therapy delivered via a mobile phone messaging robot to decrease postoperative opioid use in patients with orthopedic trauma: randomized controlled trial. Journal of medical Internet research. 2020 Jul 29;22(7):e17750. | No name | Knee fracture | mHealth |
| Ardern CL, Kvist J, BANG Trial Group Ardern Clare Kvist Joanna Fältström Anne Stålman Anders O’Halloran Paul Webster Kate Taylor Nicholas. BAck iN the Game (BANG)–a smartphone application to help athletes return to sport following anterior cruciate ligament reconstruction: protocol for a multi-centre, randomised controlled trial. BMC musculoskeletal disorders. 2020 Aug 8;21(1):523. | BANG | ACL rupture ± meniscus | mHealth |
| Kordatos G, Stavrakis M. Preliminary design of a wearable system to increase adherence to rehabilitation programmes in acute Cruciate Ligament (CL) rupture. InProceedings of the 4th EAI International Conference on Smart Objects and Technologies for Social Good 2018 Nov 28 (pp. 277-282). | No name | ACL rupture ± meniscus | mHealth |
| Bauwens PH, Fayard JM, Tatar M, Abid H, Freychet B, Sonnery-Cottet B, Thaunat M. Evaluation of a smartphone application for self-rehabilitation after anterior cruciate ligament reconstruction during a COVID-19 lockdown. Orthopaedics & Traumatology: Surgery & Research. 2023 Feb 1;109(1):103342. | Doct’up | ACL rupture ± meniscus | mHealth |
| Button K, Nicholas K, Busse M, Collins M, Spasić I. Integrating self-management support for knee injuries into routine clinical practice: TRAK intervention design and delivery. Musculoskeletal Science and Practice. 2018 Feb 1;33:53-60. | TRAK-ACL | ACL rupture | Telerehabilitation |
| Coronado RA, Sterling EK, Fenster DE, Bird ML, Heritage AJ, Woosley VL, Burston AM, Henry AL, Huston LJ, Vanston SW, Cox III CL. Cognitive-behavioral-based physical therapy to enhance return to sport after anterior cruciate ligament reconstruction: an open pilot study. Physical Therapy in Sport. 2020 Mar 1;42:82-90. | CBPT-ACLR | ACL rupture ± meniscus | Telerehabilitation |
| Dunphy E, Button K, Hamilton F, Murray E. A feasibility RCT comparing TRAK-ACL digital rehabilitation intervention plus TAU versus TAU for knee anterior cruciate ligament reconstruction patients. Physiotherapy. 2022 Feb 1;114:e73. | TRAK-ACL | ACL rupture | Telerehabilitation |
| Dunphy E, Button K, Hamilton F, Williams J, Spasic I, Murray E. Feasibility randomised controlled trial comparing TRAK-ACL digital rehabilitation intervention plus treatment as usual versus treatment as usual for patients following anterior cruciate ligament reconstruction. BMJ Open Sport & Exercise Medicine. 2021 May 6;7(2). | TRAK-ACL | ACL rupture | Telerehabilitation |
| Dunphy E, Hamilton FL, Button K. Taxonomy for the rehabilitation of knee conditions (TRAK), a digital intervention to support the self-care components of anterior cruciate ligament rehabilitation: protocol of a feasibility study. JMIR Research Protocols. 2016 Dec 5;5(4):e6402. | TRAK-ACL | ACL rupture | Telerehabilitation |
| Dunphy E, Hamilton FL, Spasić I, Button K. Acceptability of a digital health intervention alongside physiotherapy to support patients following anterior cruciate ligament reconstruction. BMC musculoskeletal disorders. 2017 Nov 21;18(1):471. | TRAK-ACL | ACL rupture | Telerehabilitation |
| Foissey C, Thaunat M, Bondoux L, Sonnery-Cottet B, Fayard JM. Self-rehabilitation guided by a mobile application after anterior cruciate ligament reconstruction leads to improved early motion and less pain. Arthroscopy, sports medicine, and rehabilitation. 2021 Oct 1;3(5):e1457-64. | Doct’up | ACL rupture ± meniscus | mHealth |
| Foissey C, Abid H, Freychet B, Sonnery-Cottet B, Thaunat M, Fayard JM. Postoperative regular use of a self-rehabilitation mobile application for more than two weeks reduces extension deficit and cyclop syndrome after anterior cruciate ligament reconstruction. Journal of Experimental Orthopaedics. 2023 Feb 9;10(1):14. | Doct’up | ACL rupture ± meniscus | mHealth |
| Galasso O, Calabrese M, Scanniello G, Garofano M, Pepe L, Budaci L, Ungaro G, Fimiani G, Bramanti P, Schiavo L, Corallo F. Accelerating recovery: a case report on telerehabilitation for a triathlete’s post-meniscus surgery comeback. InHealthcare 2025 Feb 13 (Vol. 13, No. 4, p. 406). MDPI. | Khymeia System | Non-specific knee condition | Telerehabilitation |
| Gruner MP, Hogaboom N, Hasley I, Hoffman J, Gonzalez-Carta K, Cheville AL, Li Z, Sellon JL. Prospective, single-blind, randomized controlled trial to evaluate the effectiveness of a digital exercise therapy application compared with conventional physical therapy for the treatment of nonoperative knee conditions. Archives of Rehabilitation Research and Clinical Translation. 2021 Dec 1;3(4):100151. | Limber Health DETA | Non-specific knee condition | mHealth |
| Guo Y, Li D, Wu YB, Sun X, Sun XY, Yang YP. Mobile health-based home rehabilitation education improving early outcomes after anterior cruciate ligament reconstruction: a randomized controlled clinical trial. Frontiers in Public Health. 2023 Jan 12;10:1042167. | Rehab Cloud Platform | ACL rupture ± meniscus | mhealth |
| Höher J, Lischke B, Petersen W, Mengis N, Niederer D, Stein T, Stoffels T, Prill R, Schmidt-Lucke C. Sensor-based telerehabilitation system increases patient adherence after knee surgery. PLOS Digital Health. 2023 Feb 17;2(2):e0000175. | Orthelligent | ACL rupture ± meniscus | mhealth |
| Hadley C, McGrath M, Prodoehl JP, Cohen SB, Emper WD, Hammoud S, Ciccotti MG, Freedman KB, Brahmabhatt S, Mazur DW. Comparison of traditional physical therapy to internet-based physical therapy after knee arthroscopy: a prospective randomized controlled trial comparing patient outcomes and satisfaction. Orthopaedic Journal of Sports Medicine. 2019 Jul 29;7(7_suppl5):2325967119S00344. | No name | Meniscus tear | Telerehabiltation |
| Higgins J, Semple J, Murnaghan L, Sharpe S, Theodoropoulos J. Mobile web-based follow-up for postoperative ACL reconstruction: a single-center experience. Orthopaedic Journal of Sports Medicine. 2017 Nov 21;5(12):2325967117745278. | QoC Health Inc | ACL rupture ± meniscus | mHealth |
| Higgins J, Chang J, Hoit G, Chahal J, Dwyer T, Theodoropoulos J. Conventional follow-up versus mobile application home monitoring for postoperative anterior cruciate ligament reconstruction patients: a randomized controlled trial. Arthroscopy: The Journal of Arthroscopic & Related Surgery. 2020 Jul 1;36(7):1906-16. | QoC Health Inc | ACL rupture ± meniscus | mHealth |
| Hong CK, Liu ZW, Hsu KL, Kuan FC, Yang JF, Su WR. A novel home-based rehabilitative knee brace system is a viable option for postoperative rehabilitation after anterior cruciate ligament reconstruction: a report of 15 cases. Journal of Experimental Orthopaedics. 2022 Sep 23;9(1):96. | KNEESUP | ACL rupture ± meniscus | mHealth |
| Kim G, Kim WS, Kim TW, Lee YS, Lee H, Paik NJ. Home-based rehabilitation using smart wearable knee exercise device with electrical stimulation after anterior cruciate ligament reconstruction: A study protocol for a randomized controlled trial. Medicine. 2020 May 15;99(20):e20256. | exoRehab | ACL rupture | mHealth |
| Kordatos G, Stavrakis M. Design and evaluation of a wearable system to increase adherence to rehabilitation programmes in acute cruciate ligament (CL) rupture. Multimedia Tools and Applications. 2020 Dec;79(45):33549-74. | No name | ACL rupture ± meniscus | mHealth |
| Kuenze C, Pfeiffer K, Pfeiffer M, Driban JB, Pietrosimone B. Feasibility of a wearable-based physical activity goal-setting intervention among individuals with anterior cruciate ligament reconstruction. Journal of Athletic Training. 2021 Jun 1;56(6):555-64. | Fitbit | Non-specific knee condition | mHealth |
| Kungwengwe T, Evans R. Sana: A gamified rehabilitation management system for anterior cruciate ligament reconstruction recovery. Applied Sciences. 2020 Jul 16;10(14):4868. | SANA | ACL rupture | mHealth |
| Bhachu L, Soldatova LN, Spasić I, Button K. Mobile application KneeCare to support knee rehabilitation. In2014 Science and Information Conference 2014 Aug 27 (pp. 447-451). IEEE. | KneeCare | Non-specific knee condition | mHealth |
| Lee AS, Yung PS, Ong MT, Lonsdale C, Wong TW, Siu PM, Hagger MS, Chan DK. Effectiveness of a theory-driven mHealth intervention in promoting post-surgery rehabilitation adherence in patients who had anterior cruciate ligament reconstruction: A randomized clinical trial. Social science & medicine. 2023 Oct 1;335:116219. | ACL-Well | ACL rupture ± meniscus | mHealth |
| Levinger P, Hallam K, Fraser D, Pile R, Ardern C, Moreira B, Talbot S. A novel web-support intervention to promote recovery following anterior cruciate ligament reconstruction: a pilot randomised controlled trial. Physical Therapy in Sport. 2017 Sep 1;27:29-37. | No name | ACL rupture ± meniscus | Telerehabilitation |
| Li L. Effect of remote control augmented reality multimedia technology for postoperative rehabilitation of knee joint injury. Computational and Mathematical Methods in Medicine. 2022;2022(1):9320063. | No name | Knee fracture | Telerehabilitation |
| Liao WJ, Lee KT, Chiang LY, Liang CH, Chen CP. Postoperative rehabilitation after anterior cruciate ligament reconstruction through telerehabilitation with artificial intelligence brace during COVID-19 pandemic. Journal of Clinical Medicine. 2023 Jul 24;12(14):4865. | KNEESUP | ACL rupture ± meniscus | mHealth |
| Lim JY, Yu HJ, Kim SH, Lee JI, Lim JY, Wang JH, Hwang JH. Effectiveness of in-home, augmented reality–based telerehabilitation after anterior cruciate ligament reconstruction: a randomized controlled trial. Orthopaedic Journal of Sports Medicine. 2024 Oct;12(10):23259671241271729. | UIN-CARE Home 1 | ACL rupture ± meniscus | Telerehabilitation |
| Litchfield F, Buttress S. Internet educational videos: improving functional recovery following arthroscopic meniscectomy?. Physiotherapy. 2019 Jan 1;105:e93-4. | No name | Meniscus tear | eHealth |
| Loayza BG, Bendita AC, Cjuno MH, Sulla-Torres J. Application of Kinect technology and artificial neural networks in the control of rehabilitation therapies in people with knee injuries. International Journal of Advanced Computer Science and Applications. 2020;11(8). | Microsoft Kinnect | Non-specific knee condition | Telerehabilitation |
| Ashapkina MS, Alpatov AV, Sablina VA. Online System for Involving Patients in Home-Base Rehabilitation Programs for the Knee Joint. In2020 9th Mediterranean Conference on Embedded Computing (MECO) 2020 Jun 8 (pp. 1-4). IEEE. | Nefitness | Non-specific knee condition | Telerehabilitation |
| Martin CL, Nocera M, Mercer J, Marshall SW, Davi SM, Curtin JJ, Cameron KL. Efficacy of a novel telehealth application in health behavior modification and symptomology in military service members at risk for post-traumatic osteoarthritis. Military Medicine. 2024 Sep;189(9-10):2060-8. | No name | ACL rupture or meniscus tear | mHealth |
| Mayer TA, Koska D, Harsch AK, Maiwald C. Effectiveness of self-training with a web-based digital health application versus physiotherapy in the treatment of disorders of the patella: randomized controlled trial. Journal of Medical Internet Research. 2025 May 5;27:e66463. | Mawendo | Patella dislocation | Telerehabilitation |
| Mengis N, Schmidt S, Ellermann A, Sobau C, Egloff C, Kreher MM, Ksoll K, Schmidt-Lucke C, Rippke JN. A novel sensor-based application for home-based rehabilitation can objectively measure postoperative outcomes following anterior cruciate ligament reconstruction. Journal of Personalized Medicine. 2023 Sep 19;13(9):1398. | Orthelligent | ACL rupture ± meniscus | mhealth |
| Kalofonos N, Troussas C, Krouska A, Mylonas P, Sgouropoulou C. A Personalized Fitness Application for Injury Rehabilitation Using Artificial Neural Networks. In2024 19th International Workshop on Semantic and Social Media Adaptation & Personalization (SMAP) 2024 Nov 21 (pp. 56-62). IEEE. | No name | ACL rupture ± meniscus | mhealth |
| Nguyen J, Marx R, Hidaka C, Wilson S, Lyman S. Validation of electronic administration of knee surveys among ACL‐injured patients. Knee Surgery, Sports Traumatology, Arthroscopy. 2017 Oct;25(10):3116-22. | No name | ACL rupture | eHealth |
| Olach M, Meester J, Behrens G, Giesinger J, Badulescu M, Giesinger K. Feasibility Of Web-Based Patient-Reported Outcome Assessment After Arthroscopic Knee Surgery: The Patients' Perspective. InSwiss Medical Weekly 2021 Jun 23 (Vol. 151, pp. 52S-52S). FARNSBURGERSTR 8, CH-4132 MUTTENZ, SWITZERLAND: EMH SWISS MEDICAL PUBLISHERS LTD. | CHES | Non-specific knee condition | eHealth |
| Ringberg M, Eldh AC, Ardern CL, Kvist J. Athletes’ experiences of using a self-directed psychological support, the BAck iN the Game (BANG) smartphone application, during rehabilitation for return to sports following anterior cruciate ligament reconstruction. BMC Sports Science, Medicine and Rehabilitation. 2023 Sep 19;15(1):113. | BANG | ACL rupture ± meniscus | mHealth |
| Tedesco S, Belcastro M, Torre OM, Torchia P, Alfieri D, Khokhlova L, O'Flynn B. A multi-sensors wearable system for remote assessment of physiotherapy exercises during ACL rehabilitation. In2019 26th IEEE international conference on electronics, circuits and systems (ICECS) 2019 Nov 27 (pp. 237-240). IEEE. | SKYRE | ACL rupture | mHealth |
| Tedesco S, Torre OM, Belcastro M, Torchia P, Alfieri D, Khokhlova L, Komaris SD, O’flynn B. Design of a multi-sensors wearable platform for remote monitoring of knee rehabilitation. IEEE Access. 2022 Sep 8;10:98309-28. | SKYRE | ACL rupture | mHealth |
| Schmidt S, Krahl D, Podszun J, Knecht S, Zimmerer A, Sobau C, Ellermann A, Ruhl A. Combining a digital health application with standard care significantly enhances rehabilitation outcomes for ACL surgery patients. Knee Surgery, Sports Traumatology, Arthroscopy. 2025 Apr;33(4):1241-51. | Orthopy | ACL rupture ± meniscus | mHealth |
| Sun X, Li D, Guo Y, Chu HL, Sun XY, Yang YP. Effects of mobile health intervention on early knee function after anterior cruciate ligament reconstruction: a prospective randomized controlled study. Zhonghua wai ke za zhi [Chinese Journal of Surgery]. 2020 Dec 1;58(12):936-41. | Rehab Cloud Platform | ACL rupture ± meniscus | mhealth |
| Tan EK, Hanif KA, Saaid FM, Yeak RD, Kahar JA, Mahmud A, Nasir NM, NASIR MN. Cost-Outcome Descriptive Study for Mobile App (UPSCALER) in the Rehabilitation of Anterior Cruciate Ligament (ACL) Injuries After Reconstructive Surgery. Cureus. 2024 May 8;16(5). | Upscaler | ACL rupture | mHealth |
| Tsvyakh AI, Hospodarskyy AY, Marchenkova NO, Kopytchak IR, Kostjuk VP, Lymar YA, Gdanskyi SM. Telerehabilitation of the knee joints of patients with polytrauma. Wiad Lek. 2021 Jan 1;74(1):48-51. | No name | Non-specific knee condition | Telerehabilitation |
| Vasavada KD, Shankar DS, Avila A, Mojica ES, Hurley ET, Lehane K, Buzin SD, Oeding JF, Stein SM, Gonzalez-Lomas G, Alaia MJ. Severe attrition and poor satisfaction in patients undergoing telerehabilitation vs. standard in-person rehabilitation after arthroscopic rotator cuff repairs and anterior cruciate ligament reconstructions. Surgeries. 2024 Aug 8;5(3):627-39. | Physitrack | Non-specific knee condition | Telerehabilitation |
| Wang K, Peng L, You M, Deng Q, Li J. Multicomponent supervised tele-rehabilitation versus home-based self-rehabilitation management after anterior cruciate ligament reconstruction: a study protocol for a randomized controlled trial. Journal of Orthopaedic Surgery and Research. 2024 Jun 28;19(1):381. | HJT Software | ACL rupture | mHealth |
| Wang L, Chen X, Deng Q, You M, Xu Y, Liu D, Lin Y, Li P, Li J. Effectiveness of a digital rehabilitation program based on computer vision and augmented reality for isolated meniscus injury: protocol for a prospective randomized controlled trial. Journal of Orthopaedic Surgery and Research. 2023 Dec 7;18(1):936. | No name | Meniscus tear | Telerehabilitation |
| Ling Y, Yan X. A Smart Portable Goniometer-Based Knee Rehabilitation Device Using Arduino Microprocessor and Sensors. In2022 International Conference on Manufacturing, Industrial Automation and Electronics (ICMIAE) 2022 Aug 26 (pp. 79-88). IEEE. | No name | ACL rupture ± meniscus | mHealth |
| Zadrapova M, Mrázková E, Janura M, Strycek M, Cerny M. Influence of rehabilitation aid with biofeedback on the rehabilitation process during remote home-based rehabilitation. International Journal of Environmental Research and Public Health. 2022 Jul 25;19(15):9069. | No name | ACL rupture ± meniscus | Telerehabilitation |
| Quan J Wang (2025) Comparison of the Efficacy of Telerehabilitation and In-person Rehabilitation Based on Wearable Devices After ACLR Surgery: a Multicenter Randomized Controlled Trial. Available at: https://clinicaltrials.gov/study/NCT06327555#study-overview (accessed 15 April 2026). | No name | ACL rupture | Telerehabilitation |
| Saif Usman (2016) Review of ACL Rehab app, a rehabilitation app for knee injuries. Available at: https://www.imedicalapps.com/2016/02/acl-rehab-app-knee-injuries/ (accessed 15 April 2026). | ACL Rehab | ACL rupture | mHealth |
| Richardson BR, Truter P, Blumke R, Russell TG. Physiotherapy assessment and diagnosis of musculoskeletal disorders of the knee via telerehabilitation. Journal of telemedicine and telecare. 2017 Jan;23(1):88-95. | NeoRehab | ACL rupture or meniscus tear | Telerehabilitation |
| Semple JL, Sharpe S, Murnaghan ML, Theodoropoulos J, Metcalfe KA. Using a mobile app for monitoring post-operative quality of recovery of patients at home: a feasibility study. JMIR mHealth and uHealth. 2015 Feb 12;3(1):e18. | QoC Health Inc | ACL rupture ± meniscus | mHealth |
| Aljahdali S, Latchem-Hastings J, Hamana K, Button K. EXPLORING THE PERCEPTION OF END-USERS IN INTRODUCING A SUPPORTED SELF-MANAGEMENT WEB-BASED INTERVENTION FOR KNEE CONDITIONS (TRAK) IN SAUDI ARABIA: A QUALITATIVE STUDY. Osteoarthritis and Cartilage. 2022 Apr 1;30:S386-7. | TRAK-ACL | ACL rupture | Telerehabilitation |
| Brewer BW, Van Raalte JL, Cornelius AE. An interactive cognitive-behavioural multimedia program favourably affects pain and kinesiophobia during rehabilitation after anterior cruciate ligament surgery: an effectiveness trial. International Journal of Sport and Exercise Psychology. 2022 Jul 4;20(4):1133-55. | No name | ACL rupture | eHealth |
| Spasić I, Button K, Divoli A, Gupta S, Pataky T, Pizzocaro D, Preece A, Van Deursen R, Wilson C. TRAK app suite: A web-based intervention for delivering standard care for the rehabilitation of knee conditions. JMIR research protocols. 2015 Oct 16;4(4):e4091. | TRAK-ACL | ACL rupture | Telerehabilitation |
| Tipprom A, Soontornpipit P, Sillabutra J, Satitvipawee P, Viwatwongkasem C. Development of a rehabilitation monitoring system on web-based application for patients with knee pain. In2018 International Electrical Engineering Congress (iEECON) 2018 Mar 7 (pp. 1-4). IEEE. | No name | Non-specific knee condition | Telerehabilitation |
| Truong LK, Mosewich AD, Miciak M, Gold MV, Li LC, Whittaker JL. “I FEEL EMPOWERED AND I HAVE CONTROL OVER THE SITUATION AND HOW MY KNEE IS IN THE LONG TERM.” A QUALITATIVE ANALYSIS OF THE ACCEPTABILITY OF A VIRTUAL PHYSIOTHERAPIST-GUIDED KNEE HEALTH PROGRAM FOR PERSONS AT-RISK OF KNEE OSTEOARTHRITIS. Osteoarthritis and Cartilage. 2022 Apr 1;30:S223. | SOAR | Non-specific knee condition | Telerehabilitation |
| Truong LK, Mosewich AD, Miciak M, Gold MV, Li LC, Whittaker JL. 152 ‘I feel I’m leading the charge.’A qualitative analysis of a virtual physiotherapist-guided program for persons with sport-related knee trauma. BMJ Open Sport & Exercise Medicine. 2022;8(Suppl 1):A11-. | SOAR | Non-specific knee condition | Telerehabilitation |
| Truong LK, Losciale J, Zhang K, Roos EM, Li LC, Whittaker JL. Changing Perceptions Of Knee Health Self-Management With A Virtual, Education, Action Planning And Exercise Program In People At Risk Of Post-Traumatic Osteoarthritis. Osteoarthritis and Cartilage. 2023 Mar 1;31:S200-1. | SOAR | Non-specific knee condition | Telerehabilitation |
| Zhou Yi, Zhang Xin, Wang Hongshu, Zhou Kaibo, Lu Shengdi, Xu Caiqi, Lü Yiming. Application of Digital Therapeutics in Post‑operative Rehabilitation After Anterior Cruciate Ligament Surgery. Chinese Journal of Medical Instruments. 2023 Sep 30; 47(5):487–491. | Joymotion | ACL rupture | Telerehabilitation / mHealth |
| Husselbee R, Nowak I. Evaluating impact of getUBetter, a digital self-management support tool, deployed to 14,500 patients on a community MSK physiotherapy waiting list. Physiotherapy. 2025 Apr 1;126. | GetUBetter | Non-specific knee condition | mHealth |
| Braybrooke A, Burgess R, Brooks M, Banerjee A, Hill JC. Quality indicators for the community care of MSK conditions: An online modified-Delphi study. Musculoskeletal Science and Practice. 2025 Apr 1;76:103283. | GetUBetter | Non-specific knee condition | mHealth |
| Berry A, McClellan C, Wanless B, Walsh N. A tailored app for the self-management of musculoskeletal conditions: evidencing a logic model of behavior change. JMIR Formative Research. 2022 Mar 8;6(3):e32669. | GetUBetter | Non-specific knee condition | mHealth |
| Wanless B, Hassan N, McClellan C, Sothinathan C, Abelleyra Lastoria DA, Herweijer T, Thorpe J, Noblet T, Berry A. How Do We Better Serve Excluded Populations When Delivering Digital Health Technology? Inclusion Evaluation of a Digital Musculoskeletal Self‐Management Solution. Musculoskeletal Care. 2024 Sep;22(3):e1925. | GetUBetter | Non-specific knee condition | mHealth |
| Health Innovation Network. Digital self-management for MSK conditions [Internet]. 2025. Report. Available from: https://thehealthinnovationnetwork.co.uk/case_studies/digital-self-management-for-msk-conditions/ | GetUBetter | Non-specific knee condition | mHealth |
| Bennett A, Carr M, Brunsdon R. NHS Innovation Collaborative for digital health – cross regional case study [Internet]. 2023 [cited 2026 Mar 22]. Report. Available from: https://future.nhs.uk/connect.ti/InnovationCollaborative/view?objectID=168271141 | GetUBetter | Non-specific knee condition | mHealth |
| Burgess R, Tucker K, Smithson R, Dimbleby P, Casey C. Optimising Musculoskeletal Patient Flow Through Digital Triage and Supported Self‐Management: A Service Evaluation Set Within Community Musculoskeletal Care. Musculoskeletal Care. 2024 Dec;22(4):e70013. | Phio | Non-specific knee condition | mHealth |
| Tucker K, Braybrooke A, Burgess R. Implementing a Digital Self‐Management App Can Support the NHS's Green Agenda: A Service Evaluation in a Community Musculoskeletal Physiotherapy Service. Musculoskeletal care. 2025 Jun;23(2):e70138. | Phio | Non-specific knee condition | mHealth |
| Kempton C, Ryan K, Clohessy S, Grinbergs P, Elliott MT. Capturing Objective Functional Measures Using Smartphone Inertial Sensors: Feasibility and Usability Study With Older Adults. JMIR Rehabilitation and Assistive Technologies. 2025 Sep 22;12(1):e72511. | Phio | Non-specific knee condition | mHealth |
| Morris J, O'Regan J, O'Sullivan R. Optimising patient flow through digital triage and self-management in a Musculoskeletal Service. | Phio | Non-specific knee condition | mHealth |
| Marques JF. *Consulting Project for Ageas Group Portugal Regarding the Topic:“Integration of a Remote Physical Therapy Solution” Individual Topic:“Impact Estimation of a Remote Therapy Solution Integration in Médis”* (Master's thesis, Universidade NOVA de Lisboa (Portugal)). | Sword | Non-specific knee condition | mHealth |
| Domingues B, Pereira AP, Pradhan A, Zidde C, Janela D, Marramaque C, Bento V, Yanamadala V, Cohen SP, Belz L, Wang K. Digital Versus In-Person Physical Therapy in Adults With Musculoskeletal Conditions: Retrospective Matched-Cohort Analysis of Surgery and Low-Value Surgical Rates. Journal of medical Internet research. 2025 Dec 17;27:e82573. | Sword | Non-specific knee condition | mHealth |
| Pereira AP, Janela D, Areias AC, Molinos M, Tong X, Bento V, Yanamadala V, Cohen SP, Correia FD, Costa F. Evaluating digital rehabilitation outcomes in chronic musculoskeletal conditions across non-obesity, obesity, and severe obesity. Journal of Pain Research. 2025 Dec 31:73-87. | Sword | Non-specific knee condition | mHealth |
| Areias AC, Janela D, Molinos M, Moulder RG, Bento V, Yanamadala V, Cohen SP, Correia FD, Costa F. Managing musculoskeletal pain in older adults through a digital care solution: secondary analysis of a prospective clinical study. JMIR Rehabilitation and Assistive Technologies. 2023 Aug 15;10:e49673. | Sword | Non-specific knee condition | mHealth |
| Areias AC, Janela D, Molinos M, Bento V, Moreira C, Yanamadala V, Cohen SP, Correia FD, Costa F. Exploring the importance of race and gender concordance between patients and physical therapists in digital rehabilitation for musculoskeletal conditions: Observational, longitudinal study. Journal of Medical Internet Research. 2024 Oct 29;26:e65354. | Sword | Non-specific knee condition | mHealth |
| Scheer J, Costa F, Molinos M, Areias A, Janela D, Moulder RG, Lains J, Bento V, Yanamadala V, Cohen SP, Correia FD. Racial and ethnic differences in outcomes of a 12-week digital rehabilitation program for musculoskeletal pain: prospective longitudinal cohort study. Journal of medical Internet research. 2022 Oct 31;24(10):e41306. | Sword | Non-specific knee condition | mHealth |
| Areias AC, Doverspike D, Brostek DF, Janela D, Erwin MS, Pinter JM, Ficke JR, Costa F. Transforming veteran rehabilitation care: learnings from a remote digital approach for musculoskeletal pain. InHealthcare 2024 Jul 31 (Vol. 12, No. 15, p. 1518). MDPI. | Sword | Non-specific knee condition | mHealth |
